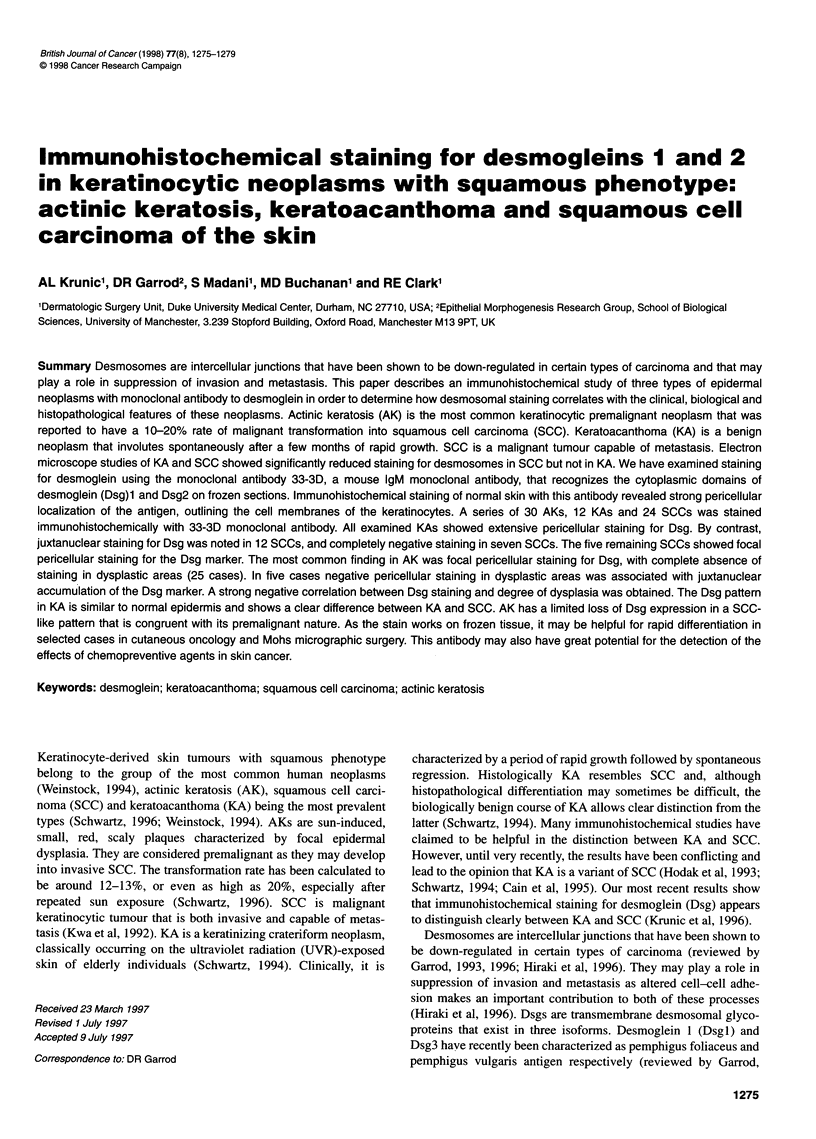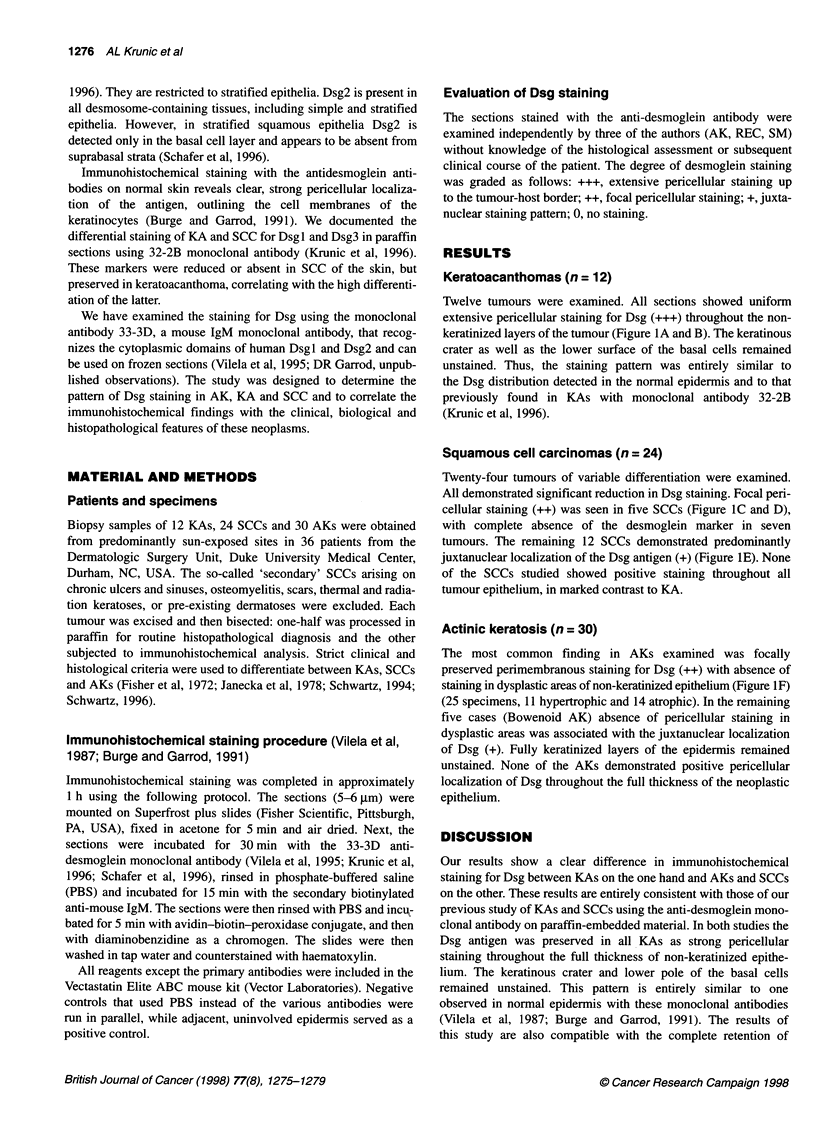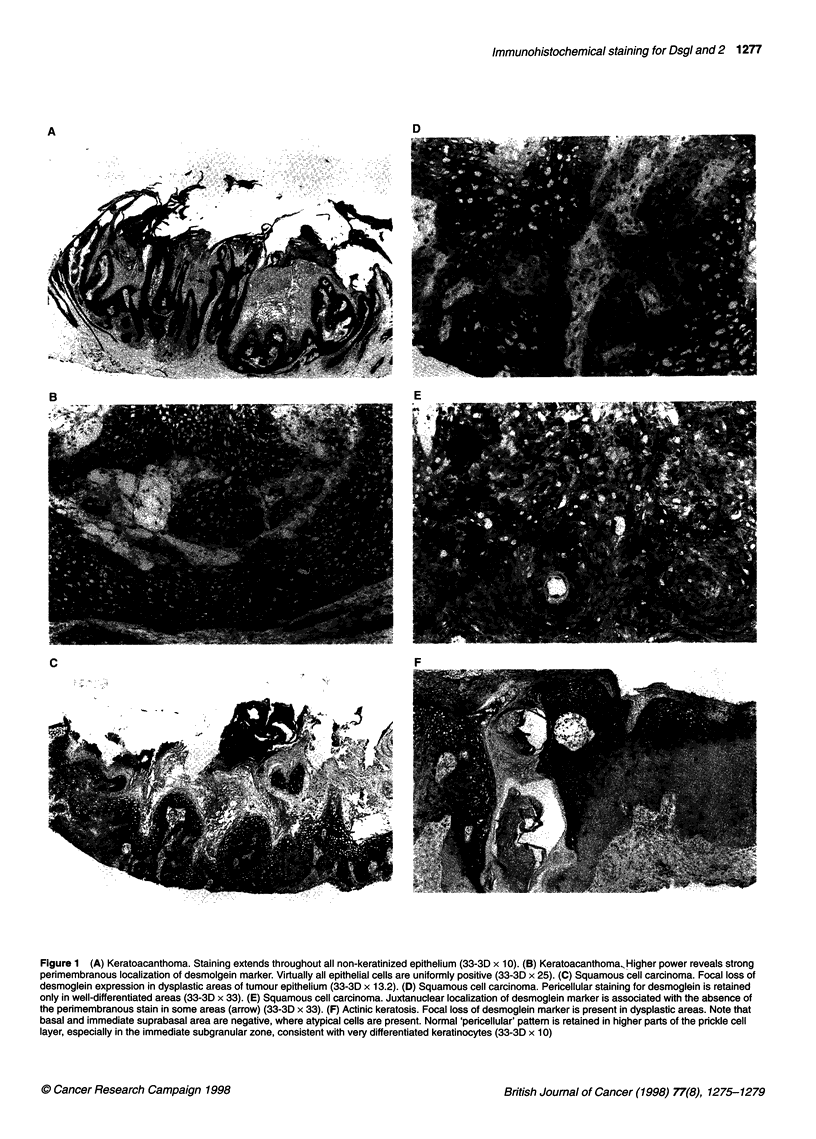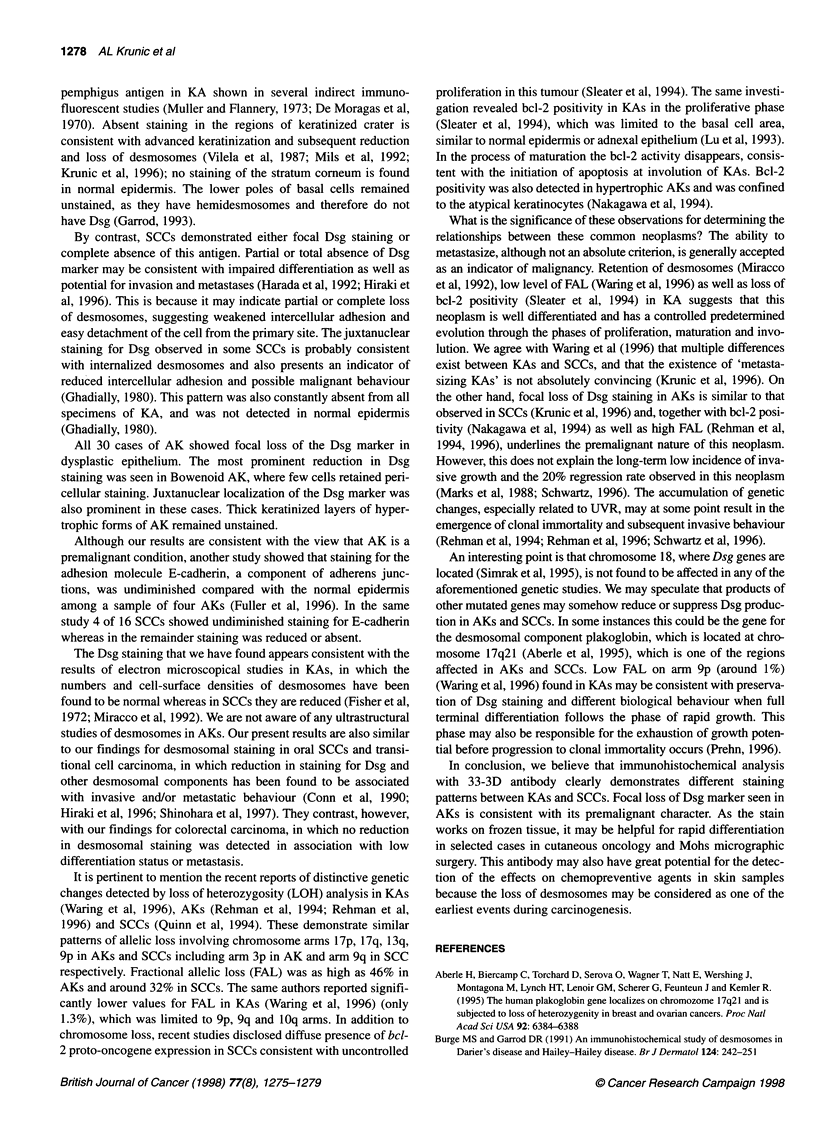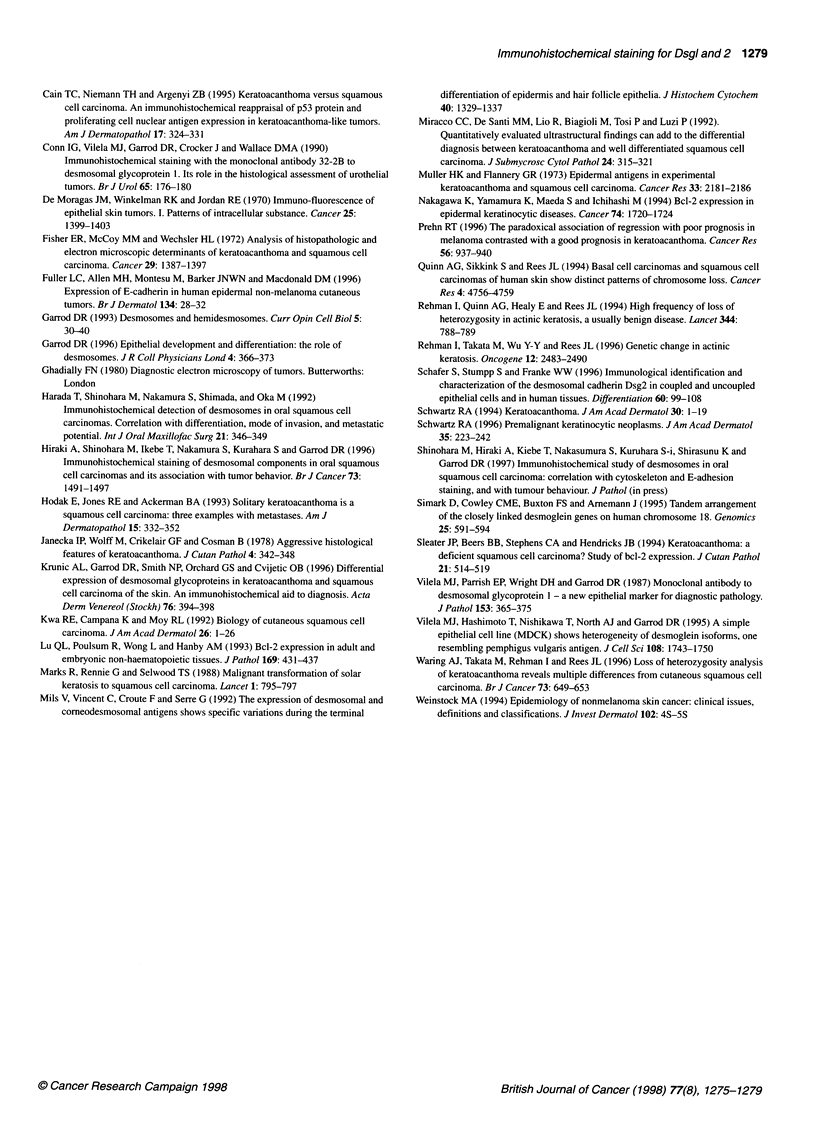# Immunohistochemical staining for desmogleins 1 and 2 in keratinocytic neoplasms with squamous phenotype: actinic keratosis, keratoacanthoma and squamous cell carcinoma of the skin.

**DOI:** 10.1038/bjc.1998.213

**Published:** 1998-04

**Authors:** A. L. Krunic, D. R. Garrod, S. Madani, M. D. Buchanan, R. E. Clark

**Affiliations:** Dermatologic Surgery Unit, Duke University Medical Center, Durham, NC 27710, USA.

## Abstract

**Images:**


British Journal of Cancer (1998) 77(8), 1275–1279
© 1998 Cancer Research Campaign

### Immunohistochemical staining for desmogleins 1 and 2 in keratinocytic neoplasms with squamous phenotype: actinic keratosis, keratoacanthoma and squamous cell carcinoma of the skin

**AL Krunic[1], DR Garrod[2], S Madani[1], MD Buchanan[1] and RE Clark[1]**

[1]Dermatologic Surgery Unit, Duke University Medical Center, Durham, NC 27710, USA; [2]Epithelial Morphogenesis Research Group, School of Biological Sciences, University of Manchester, 3.239 Stopford Building, Oxford Road, Manchester M13 9PT, UK

**Summary** Desmosomes are intercellular junctions that have been shown to be down-regulated in certain types of carcinoma and that may play a role in suppression of invasion and metastasis. This paper describes an immunohistochemical study of three types of epidermal neoplasms with monoclonal antibody to desmoglein in order to determine how desmosomal staining correlates with the clinical, biological and histopathological features of these neoplasms. Actinic keratosis (AK) is the most common keratinocytic premalignant neoplasm that was reported to have a 10–20% rate of malignant transformation into squamous cell carcinoma (SCC). Keratoacanthoma (KA) is a benign neoplasm that involutes spontaneously after a few months of rapid growth. SCC is a malignant tumour capable of metastasis. Electron microscope studies of KA and SCC showed significantly reduced staining for desmosomes in SCC but not in KA. We have examined staining for desmoglein using the monoclonal antibody 33-3D, a mouse IgM monoclonal antibody, that recognizes the cytoplasmic domains of desmoglein (Dsg)1 and Dsg2 on frozen sections. Immunohistochemical staining of normal skin with this antibody revealed strong pericellular localization of the antigen, outlining the cell membranes of the keratinocytes. A series of 30 AKs, 12 KAs and 24 SCCs was stained immunohistochemically with 33-3D monoclonal antibody. All examined KAs showed extensive pericellular staining for Dsg. By contrast, juxtanuclear staining for Dsg was noted in 12 SCCs, and completely negative staining in seven SCCs. The five remaining SCCs showed focal pericellular staining for the Dsg marker. The most common finding in AK was focal pericellular staining for Dsg, with complete absence of staining in dysplastic areas (25 cases). In five cases negative pericellular staining in dysplastic areas was associated with juxtanuclear accumulation of the Dsg marker. A strong negative correlation between Dsg staining and degree of dysplasia was obtained. The Dsg pattern in KA is similar to normal epidermis and shows a clear difference between KA and SCC. AK has a limited loss of Dsg expression in a SCC-like pattern that is congruent with its premalignant nature. As the stain works on frozen tissue, it may be helpful for rapid differentiation in selected cases in cutaneous oncology and Mohs micrographic surgery. This antibody may also have great potential for the detection of the effects of chemopreventive agents in skin cancer.

**Keywords:** desmoglein; keratoacanthoma; squamous cell carcinoma; actinic keratosis

Keratinocyte-derived skin tumours with squamous phenotype belong to the group of the most common human neoplasms (Weinstock, 1994), actinic keratosis (AK), squamous cell carcinoma (SCC) and keratoacanthoma (KA) being the most prevalent types (Schwartz, 1996; Weinstock, 1994). AKs are sun-induced, small, red, scaly plaques characterized by focal epidermal dysplasia. They are considered premalignant as they may develop into invasive SCC. The transformation rate has been calculated to be around 12–13%, or even as high as 20%, especially after repeated sun exposure (Schwartz, 1996). SCC is malignant keratinocytic tumour that is both invasive and capable of metastasis (Kwa et al, 1992). KA is a keratinizing crateriform neoplasm, classically occurring on the ultraviolet radiation (UVR)-exposed skin of elderly individuals (Schwartz, 1994). Clinically, it is characterized by a period of rapid growth followed by spontaneous regression. Histologically KA resembles SCC and, although histopathological differentiation may sometimes be difficult, the biologically benign course of KA allows clear distinction from the latter (Schwartz, 1994). Many immunohistochemical studies have claimed to be helpful in the distinction between KA and SCC. However, until very recently, the results have been conflicting and lead to the opinion that KA is a variant of SCC (Hodak et al, 1993; Schwartz, 1994; Cain et al, 1995). Our most recent results show that immunohistochemical staining for desmoglein (Dsg) appears to distinguish clearly between KA and SCC (Krunic et al, 1996).

*Received 23 March 1997*
*Revised 1 July 1997*
*Accepted 9 July 1997*

*Correspondence to:* DR Garrod

Desmosomes are intercellular junctions that have been shown to be down-regulated in certain types of carcinoma (reviewed by Garrod, 1993, 1996; Hiraki et al, 1996). They may play a role in suppression of invasion and metastasis as altered cell–cell adhesion makes an important contribution to both of these processes (Hiraki et al, 1996). Dsgs are transmembrane desmosomal glycoproteins that exist in three isoforms. Desmoglein 1 (Dsg1) and Dsg3 have recently been characterized as pemphigus foliaceus and pemphigus vulgaris antigen respectively (reviewed by Garrod,

1996). They are restricted to stratified epithelia. Dsg2 is present in all desmosome-containing tissues, including simple and stratified epithelia. However, in stratified squamous epithelia Dsg2 is detected only in the basal cell layer and appears to be absent from suprabasal strata (Schafer et al, 1996).

Immunohistochemical staining with the antidesmoglein antibodies on normal skin reveals clear, strong pericellular localization of the antigen, outlining the cell membranes of the keratinocytes (Burge and Garrod, 1991). We documented the differential staining of KA and SCC for Dsg1 and Dsg3 in paraffin sections using 32-2B monoclonal antibody (Krunic et al, 1996). These markers were reduced or absent in SCC of the skin, but preserved in keratoacanthoma, correlating with the high differentiation of the latter.

We have examined the staining for Dsg using the monoclonal antibody 33-3D, a mouse IgM monoclonal antibody, that recognizes the cytoplasmic domains of human Dsg1 and Dsg2 and can be used on frozen sections (Vilela et al, 1995; DR Garrod, unpublished observations). The study was designed to determine the pattern of Dsg staining in AK, KA and SCC and to correlate the immunohistochemical findings with the clinical, biological and histopathological features of these neoplasms.

### MATERIAL AND METHODS

### Patients and specimens

Biopsy samples of 12 KAs, 24 SCCs and 30 AKs were obtained from predominantly sun-exposed sites in 36 patients from the Dermatologic Surgery Unit, Duke University Medical Center, Durham, NC, USA. The so-called 'secondary' SCCs arising on chronic ulcers and sinuses, osteomyelitis, scars, thermal and radiation keratoses, or pre-existing dermatoses were excluded. Each tumour was excised and then bisected: one-half was processed in paraffin for routine histopathological diagnosis and the other subjected to immunohistochemical analysis. Strict clinical and histological criteria were used to differentiate between KAs, SCCs and AKs (Fisher et al, 1972; Janecka et al, 1978; Schwartz, 1994; Schwartz, 1996).

### Immunohistochemical staining procedure (Vilela et al, 1987; Burge and Garrod, 1991)

Immunohistochemical staining was completed in approximately 1 h using the following protocol. The sections (5–6 μm) were mounted on Superfrost plus slides (Fisher Scientific, Pittsburgh, PA, USA), fixed in acetone for 5 min and air dried. Next, the sections were incubated for 30 min with the 33-3D anti-desmoglein monoclonal antibody (Vilela et al, 1995; Krunic et al, 1996; Schafer et al, 1996), rinsed in phosphate-buffered saline (PBS) and incubated for 15 min with the secondary biotinylated anti-mouse IgM. The sections were then rinsed with PBS and incubated for 5 min with avidin–biotin–peroxidase conjugate, and then with diaminobenzidine as a chromogen. The slides were then washed in tap water and counterstained with haematoxylin.

All reagents except the primary antibodies were included in the Vectastatin Elite ABC mouse kit (Vector Laboratories). Negative controls that used PBS instead of the various antibodies were run in parallel, while adjacent, uninvolved epidermis served as a positive control.

### Evaluation of Dsg staining

The sections stained with the anti-desmoglein antibody were examined independently by three of the authors (AK, REC, SM) without knowledge of the histological assessment or subsequent clinical course of the patient. The degree of desmoglein staining was graded as follows: +++, extensive pericellular staining up to the tumour-host border; ++, focal pericellular staining; +, juxtanuclear staining pattern; 0, no staining.

### RESULTS

### Keratoacanthomas (*n* = 12)

Twelve tumours were examined. All sections showed uniform extensive pericellular staining for Dsg (+++) throughout the non-keratinized layers of the tumour (Figure 1A and B). The keratinous crater as well as the lower surface of the basal cells remained unstained. Thus, the staining pattern was entirely similar to the Dsg distribution detected in the normal epidermis and to that previously found in KAs with monoclonal antibody 32-2B (Krunic et al, 1996).

### Squamous cell carcinomas (*n* = 24)

Twenty-four tumours of variable differentiation were examined. All demonstrated significant reduction in Dsg staining. Focal pericellular staining (++) was seen in five SCCs (Figure 1C and D), with complete absence of the desmoglein marker in seven tumours. The remaining 12 SCCs demonstrated predominantly juxtanuclear localization of the Dsg antigen (+) (Figure 1E). None of the SCCs studied showed positive staining throughout all tumour epithelium, in marked contrast to KA.

### Actinic keratosis (*n* = 30)

The most common finding in AKs examined was focally preserved perimembranous staining for Dsg (++) with absence of staining in dysplastic areas of non-keratinized epithelium (Figure 1F) (25 specimens, 11 hypertrophic and 14 atrophic). In the remaining five cases (Bowenoid AK) absence of pericellular staining in dysplastic areas was associated with the juxtanuclear localization of Dsg (+). Fully keratinized layers of the epidermis remained unstained. None of the AKs demonstrated positive pericellular localization of Dsg throughout the full thickness of the neoplastic epithelium.

### DISCUSSION

Our results show a clear difference in immunohistochemical staining for Dsg between KAs on the one hand and AKs and SCCs on the other. These results are entirely consistent with those of our previous study of KAs and SCCs using the anti-desmoglein monoclonal antibody on paraffin-embedded material. In both studies the Dsg antigen was preserved in all KAs as strong pericellular staining throughout the full thickness of non-keratinized epithelium. The keratinous crater and lower pole of the basal cells remained unstained. This pattern is entirely similar to one observed in normal epidermis with these monoclonal antibodies (Vilela et al, 1987; Burge and Garrod, 1991). The results of this study are also compatible with the complete retention of

© Cancer Research Campaign 1998

**Figure 1** (**A**) Keratoacanthoma. Staining extends throughout all non-keratinized epithelium (33-3D × 10). (**B**) Keratoacanthoma. Higher power reveals strong perimembranous localization of desmolgein marker. Virtually all epithelial cells are uniformly positive (33-3D × 25). (**C**) Squamous cell carcinoma. Focal loss of desmoglein expression in dysplastic areas of tumour epithelium (33-3D × 13.2). (**D**) Squamous cell carcinoma. Pericellular staining for desmoglein is retained only in well-differentiated areas (33-3D × 33). (**E**) Squamous cell carcinoma. Juxtanuclear localization of desmoglein marker is associated with the absence of the perimembranous stain in some areas (arrow) (33-3D × 33). (**F**) Actinic keratosis. Focal loss of desmoglein marker is present in dysplastic areas. Note that basal and immediate suprabasal area are negative, where atypical cells are present. Normal 'pericellular' pattern is retained in higher parts of the prickle cell layer, especially in the immediate subgranular zone, consistent with very differentiated keratinocytes (33-3D × 10)

pemphigus antigen in KA shown in several indirect immunofluorescent studies (Muller and Flannery, 1973; De Moragas et al, 1970). Absent staining in the regions of keratinized crater is consistent with advanced keratinization and subsequent reduction and loss of desmosomes (Vilela et al, 1987; Mils et al, 1992; Krunic et al, 1996); no staining of the stratum corneum is found in normal epidermis. The lower poles of basal cells remained unstained, as they have hemidesmosomes and therefore do not have Dsg (Garrod, 1993).

By contrast, SCCs demonstrated either focal Dsg staining or complete absence of this antigen. Partial or total absence of Dsg marker may be consistent with impaired differentiation as well as potential for invasion and metastases (Harada et al, 1992; Hiraki et al, 1996). This is because it may indicate partial or complete loss of desmosomes, suggesting weakened intercellular adhesion and easy detachment of the cell from the primary site. The juxtanuclear staining for Dsg observed in some SCCs is probably consistent with internalized desmosomes and also presents an indicator of reduced intercellular adhesion and possible malignant behaviour (Ghadially, 1980). This pattern was also constantly absent from all specimens of KA, and was not detected in normal epidermis (Ghadially, 1980).

All 30 cases of AK showed focal loss of the Dsg marker in dysplastic epithelium. The most prominent reduction in Dsg staining was seen in Bowenoid AK, where few cells retained pericellular staining. Juxtanuclear localization of the Dsg marker was also prominent in these cases. Thick keratinized layers of hypertrophic forms of AK remained unstained.

Although our results are consistent with the view that AK is a premalignant condition, another study showed that staining for the adhesion molecule E-cadherin, a component of adherens junctions, was undiminished compared with the normal epidermis among a sample of four AKs (Fuller et al, 1996). In the same study 4 of 16 SCCs showed undiminished staining for E-cadherin whereas in the remainder staining was reduced or absent.

The Dsg staining that we have found appears consistent with the results of electron microscopical studies in KAs, in which the numbers and cell-surface densities of desmosomes have been found to be normal whereas in SCCs they are reduced (Fisher et al, 1972; Miracco et al, 1992). We are not aware of any ultrastructural studies of desmosomes in AKs. Our present results are also similar to our findings for desmosomal staining in oral SCCs and transitional cell carcinoma, in which reduction in staining for Dsg and other desmosomal components has been found to be associated with invasive and/or metastatic behaviour (Conn et al, 1990; Hiraki et al, 1996; Shinohara et al, 1997). They contrast, however, with our findings for colorectal carcinoma, in which no reduction in desmosomal staining was detected in association with low differentiation status or metastasis.

It is pertinent to mention the recent reports of distinctive genetic changes detected by loss of heterozygosity (LOH) analysis in KAs (Waring et al, 1996), AKs (Rehman et al, 1994; Rehman et al, 1996) and SCCs (Quinn et al, 1994). These demonstrate similar patterns of allelic loss involving chromosome arms 17p, 17q, 13q, 9p in AKs and SCCs including arm 3p in AK and arm 9q in SCC respectively. Fractional allelic loss (FAL) was as high as 46% in AKs and around 32% in SCCs. The same authors reported significantly lower values for FAL in KAs (Waring et al, 1996) (only 1.3%), which was limited to 9p, 9q and 10q arms. In addition to chromosome loss, recent studies disclosed diffuse presence of *bcl-2* proto-oncogene expression in SCCs consistent with uncontrolled proliferation in this tumour (Sleater et al, 1994). The same investigation revealed bcl-2 positivity in KAs in the proliferative phase (Sleater et al, 1994), which was limited to the basal cell area, similar to normal epidermis or adnexal epithelium (Lu et al, 1993). In the process of maturation the bcl-2 activity disappears, consistent with the initiation of apoptosis at involution of KAs. Bcl-2 positivity was also detected in hypertrophic AKs and was confined to the atypical keratinocytes (Nakagawa et al, 1994).

What is the significance of these observations for determining the relationships between these common neoplasms? The ability to metastasize, although not an absolute criterion, is generally accepted as an indicator of malignancy. Retention of desmosomes (Miracco et al, 1992), low level of FAL (Waring et al, 1996) as well as loss of bcl-2 positivity (Sleater et al, 1994) in KA suggests that this neoplasm is well differentiated and has a controlled predetermined evolution through the phases of proliferation, maturation and involution. We agree with Waring et al (1996) that multiple differences exist between KAs and SCCs, and that the existence of 'metastasizing KAs' is not absolutely convincing (Krunic et al, 1996). On the other hand, focal loss of Dsg staining in AKs is similar to that observed in SCCs (Krunic et al, 1996) and, together with bcl-2 positivity (Nakagawa et al, 1994) as well as high FAL (Rehman et al, 1994, 1996), underlines the premalignant nature of this neoplasm. However, this does not explain the long-term low incidence of invasive growth and the 20% regression rate observed in this neoplasm (Marks et al, 1988; Schwartz, 1996). The accumulation of genetic changes, especially related to UVR, may at some point result in the emergence of clonal immortality and subsequent invasive behaviour (Rehman et al, 1994; Rehman et al, 1996; Schwartz et al, 1996).

An interesting point is that chromosome 18, where *Dsg* genes are located (Simrak et al, 1995), is not found to be affected in any of the aforementioned genetic studies. We may speculate that products of other mutated genes may somehow reduce or suppress Dsg production in AKs and SCCs. In some instances this could be the gene for the desmosomal component plakoglobin, which is located at chromosome 17q21 (Aberle et al, 1995), which is one of the regions affected in AKs and SCCs. Low FAL on arm 9p (around 1%) (Waring et al, 1996) found in KAs may be consistent with preservation of Dsg staining and different biological behaviour when full terminal differentiation follows the phase of rapid growth. This phase may also be responsible for the exhaustion of growth potential before progression to clonal immortality occurs (Prehn, 1996).

In conclusion, we believe that immunohistochemical analysis with 33-3D antibody clearly demonstrates different staining patterns between KAs and SCCs. Focal loss of Dsg marker seen in AKs is consistent with its premalignant character. As the stain works on frozen tissue, it may be helpful for rapid differentiation in selected cases in cutaneous oncology and Mohs micrographic surgery. This antibody may also have great potential for the detection of the effects on chemopreventive agents in skin samples because the loss of desmosomes may be considered as one of the earliest events during carcinogenesis.

### REFERENCES

Aberle H, Biercamp C, Torchard D, Serova O, Wagner T, Natt E, Wershing J, Montagona M, Lynch HT, Lenoir GM, Scherer G, Feunteun J and Kemler R. (1995) The human plakoglobin gene localizes on chromozome 17q21 and is subjected to loss of heterozygenity in breast and ovarian cancers. *Proc Natl Acad Sci USA* **92**: 6384–6388

Burge MS and Garrod DR (1991) An immunohistochemical study of desmosomes in Darier's disease and Hailey–Hailey disease. *Br J Dermatol* **124**: 242–251

*© Cancer Research Campaign 1998*

Cain TC, Niemann TH and Argenyi ZB (1995) Keratoacanthoma versus squamous cell carcinoma. An immunohistochemical reappraisal of p53 protein and proliferating cell nuclear antigen expression in keratoacanthoma-like tumors. *Am J Dermatopathol* **17**: 324–331

Conn IG, Vilela MJ, Garrod DR, Crocker J and Wallace DMA (1990) Immunohistochemical staining with the monoclonal antibody 32-2B to desmosomal glycoprotein 1. Its role in the histological assessment of urothelial tumors. *Br J Urol* **65**: 176–180

De Moragas JM, Winkelman RK and Jordan RE (1970) Immuno-fluorescence of epithelial skin tumors. I. Patterns of intracellular substance. *Cancer* **25**: 1399–1403

Fisher ER, McCoy MM and Wechsler HL (1972) Analysis of histopathologic and electron microscopic determinants of keratoacanthoma and squamous cell carcinoma. *Cancer* **29**: 1387–1397

Fuller LC, Allen MH, Montesu M, Barker JNWN and Macdonald DM (1996) Expression of E-cadherin in human epidermal non-melanoma cutaneous tumors. *Br J Dermatol* **134**: 28–32

Garrod DR (1993) Desmosomes and hemidesmosomes. *Curr Opin Cell Biol* **5**: 30–40

Garrod DR (1996) Epithelial development and differentiation: the role of desmosomes. *J R Coll Physicians Lond* **4**: 366–373

Ghadially FN (1980) Diagnostic electron microscopy of tumors. Butterworths: London

Harada T, Shinohara M, Nakamura S, Shimada, and Oka M (1992) Immunohistochemical detection of desmosomes in oral squamous cell carcinomas. Correlation with differentiation, mode of invasion, and metastatic potential. *Int J Oral Maxillofac Surg* **21**: 346–349

Hiraki A, Shinohara M, Ikebe T, Nakamura S, Kurahara S and Garrod DR (1996) Immunohistochemical staining of desmosomal components in oral squamous cell carcinomas and its association with tumor behavior. *Br J Cancer* **73**: 1491–1497

Hodak E, Jones RE and Ackerman BA (1993) Solitary keratoacanthoma is a squamous cell carcinoma: three examples with metastases. *Am J Dermatopathol* **15**: 332–352

Janecka IP, Wolff M, Crikelair GF and Cosman B (1978) Aggressive histological features of keratoacanthoma. *J Cutan Pathol* **4**: 342–348

Krunic AL, Garrod DR, Smith NP, Orchard GS and Cvijetic OB (1996) Differential expression of desmosomal glycoproteins in keratoacanthoma and squamous cell carcinoma of the skin. An immunohistochemical aid to diagnosis. *Acta Derm Venereol (Stockh)* **76**: 394–398

Kwa RE, Campana K and Moy RL (1992) Biology of cutaneous squamous cell carcinoma. *J Am Acad Dermatol* **26**: 1–26

Lu QL, Poulsum R, Wong L and Hanby AM (1993) Bcl-2 expression in adult and embryonic non-haematopoietic tissues. *J Pathol* **169**: 431–437

Marks R, Rennie G and Selwood TS (1988) Malignant transformation of solar keratosis to squamous cell carcinoma. *Lancet* **1**: 795–797

Mils V, Vincent C, Croute F and Serre G (1992) The expression of desmosomal and corneodesmosomal antigens shows specific variations during the terminal differentiation of epidermis and hair follicle epithelia. *J Histochem Cytochem* **40**: 1329–1337

Miracco CC, De Santi MM, Lio R, Biagioli M, Tosi P and Luzi P (1992). Quantitatively evaluated ultrastructural findings can add to the differential diagnosis between keratoacanthoma and well differentiated squamous cell carcinoma. *J Submycrosc Cytol Pathol* **24**: 315–321

Muller HK and Flannery GR (1973) Epidermal antigens in experimental keratoacanthoma and squamous cell carcinoma. *Cancer Res* **33**: 2181–2186

Nakagawa K, Yamamura K, Maeda S and Ichihashi M (1994) Bcl-2 expression in epidermal keratinocytic diseases. *Cancer* **74**: 1720–1724

Prehn RT (1996) The paradoxical association of regression with poor prognosis in melanoma contrasted with a good prognosis in keratoacanthoma. *Cancer Res* **56**: 937–940

Quinn AG, Sikkink S and Rees JL (1994) Basal cell carcinomas and squamous cell carcinomas of human skin show distinct patterns of chromosome loss. *Cancer Res* **4**: 4756–4759

Rehman I, Quinn AG, Healy E and Rees JL (1994) High frequency of loss of heterozygosity in actinic keratosis, a usually benign disease. *Lancet* **344**: 788–789

Rehman I, Takata M, Wu Y-Y and Rees JL (1996) Genetic change in actinic keratosis. *Oncogene* **12**: 2483–2490

Schafer S, Stumpp S and Franke WW (1996) Immunological identification and characterization of the desmosomal cadherin Dsg2 in coupled and uncoupled epithelial cells and in human tissues. *Differentiation* **60**: 99–108

Schwartz RA (1994) Keratoacanthoma. *J Am Acad Dermatol* **30**: 1–19

Schwartz RA (1996) Premalignant keratinocytic neoplasms. *J Am Acad Dermatol* **35**: 223–242

Shinohara M, Hiraki A, Kiebe T, Nakasumura S, Kuruhara S-i, Shirasunu K and Garrod DR (1997) Immunohistochemical study of desmosomes in oral squamous cell carcinoma: correlation with cytoskeleton and E-adhesion staining, and with tumour behaviour. *J Pathol* (in press)

Simark D, Cowley CME, Buxton FS and Arnemann J (1995) Tandem arrangement of the closely linked desmoglein genes on human chromosome 18. *Genomics* **25**: 591–594

Sleater JP, Beers BB, Stephens CA and Hendricks JB (1994) Keratoacanthoma: a deficient squamous cell carcinoma? Study of bcl-2 expression. *J Cutan Pathol* **21**: 514–519

Vilela MJ, Parrish EP, Wright DH and Garrod DR (1987) Monoclonal antibody to desmosomal glycoprotein 1 – a new epithelial marker for diagnostic pathology. *J Pathol* **153**: 365–375

Vilela MJ, Hashimoto T, Nishikawa T, North AJ and Garrod DR (1995) A simple epithelial cell line (MDCK) shows heterogeneity of desmoglein isoforms, one resembling pemphigus vulgaris antigen. *J Cell Sci* **108**: 1743–1750

Waring AJ, Takata M, Rehman I and Rees JL (1996) Loss of heterozygosity analysis of keratoacanthoma reveals multiple differences from cutaneous squamous cell carcinoma. *Br J Cancer* **73**: 649–653

Weinstock MA (1994) Epidemiology of nonmelanoma skin cancer: clinical issues, definitions and classifications. *J Invest Dermatol* **102**: 4S–5S

© Cancer Research Campaign 1998